# The Effect of Standard Concentration Infusions on Medication Errors in Neonatal and Pediatric Healthcare Settings: A Systematic Review

**DOI:** 10.3390/jcm14227965

**Published:** 2025-11-10

**Authors:** Lisa Wende, Mark Schoberer, Almuth Kaune, Karen B. Kreutzer, Thorsten Orlikowsky, Nanna Christiansen, Per Nydert, Sebastian Schubert, Albrecht Eisert

**Affiliations:** 1 Hospital Pharmacy, RWTH Aachen University Hospital, 52074 Aachen, Germany; lwende@ukaachen.de; 2 Section of Neonatology, Department of Pediatric and Adolescent Medicine, RWTH Aachen University Hospital, 52074 Aachen, Germany; mschoberer@ukaachen.de (M.S.); torlikowsky@ukaachen.de (T.O.); 3 Hospital Pharmacy, University Hospital Carl Gustav Carus, TU Dresden, 01307 Dresden, Germany; almuth.kaune@ukdd.de; 4 Department of Neonatology, University Children’s Hospital Tübingen, 72076 Tübingen, Germany; karen.kreutzer@med.uni-tuebingen.de; 5 Pharmacy Department, Evelina London Children’s Hospital, London SE1 7EH, UK; n.christiansen@nhs.net; 6 Pediatric Drug Therapy Group, Astrid Lindgren Children’s Hospital, 171 76 Stockholm, Sweden; per.nydert@regionstockholm.se; 7 Hospital Pharmacy, Medical Center of the Johannes Gutenberg University Mainz, 55131 Mainz, Germany; sebastian.schubert@unimedizin-mainz.de; 8 Institute of Clinical Pharmacology, RWTH Aachen University Hospital, 52074 Aachen, Germany

**Keywords:** standard concentration, pediatric intensive care, neonatal intensive care, medication safety, medication error, error rate, parenteral medication, drug preparation

## Abstract

**Background and Objectives**: Neonates and children are at high risk of medication errors (ME) with intravenous (IV) infusion therapies, particularly when strategies such as the “rule of six” with individualized, weight-based concentrations are used. Standard concentrations (SCs) have been proposed to reduce variability, improve safety, and facilitate the use of electronic prescribing and smart infusion technologies. The impact of SCs for continuous IV infusions on ME rates in neonatal and pediatric settings was systematically evaluated. **Methods**: A systematic review was conducted in MEDLINE, Embase and CINAHL (database inception-August 2025) following Preferred Reporting Items for Systematic Reviews and Meta-Analyses (PRISMA) guidelines. Studies implementing SCs for neonatal or pediatric IV infusions and measuring outcomes referring to ME rates were included. The relative risk reduction/increase in MEs was calculated. **Results**: Five uncontrolled before-after studies published between 2005 and 2020 were included. The relative risk reductions ranged from 41.2% to 95.1% for overall ME and from 49.7% to 100% for selected error types. In two studies, the relative risk for two error types (paper-generated prescriptions, administration) increased after implementing SCs. Reviewed benefits included decreased calculation and preparation errors, reduced medication process times, improved workflow efficiency and high staff satisfaction. **Conclusions**: SCs may contribute to safer IV infusion practice in the neonatal and pediatric setting, especially combined with smart technologies and training. However, current evidence is limited and heterogeneous. Larger, prospective experimental studies are needed to confirm their long-term impact on pediatric ME rates.

## 1. Introduction

Pediatric inpatients, especially neonates in neonatal intensive care units (NICUs), are at increased risk of harm due to adverse drug events (ADEs), which are often linked to medication errors (MEs) [1,2,3,4,5]. Critically ill children often receive intravenous (IV) medications classified as “high-risk”, which have a narrow therapeutic safety margin and require precise dosing to avoid serious clinical consequences [6]. Various studies identified drug reconstitution and administration as the most error-prone steps in the medication process, particularly due to the need for several serial dilutions when no commercially available drug concentration exists, confirming low accuracy in prepared concentrations [7,8,9,10]. Other studies reported that MEs most frequently occur during the prescribing and administration process [1,3,4,5,11,12]. Common errors include calculation mistakes, knowledge deficits, misidentification, labeling errors and incorrect drug preparation [2,13,14]. Systematic reviews by Koeck et al. highlight targeted interventions in prescribing, dispensing, administration, and monitoring significantly reducing MEs [11,12]. Other studies have shown that centralized preparation, for example, in a hospital pharmacy, improves preparation accuracy and reduces the risk of antimicrobial contamination [15,16]. Increasing attention has been given to standardizing medication processes as a strategy to reduce MEs and enhance patient safety. Various approaches have been introduced, ranging from standardization of dosing units [17], formularies [18] and preparation process [19] to doses per weight range [20].

Continuous IV infusions in pediatric and neonatal settings are often administered using individualized, weight-based concentrations. Typical drug preparation on wards involves multiple manual calculations and manipulations [5,9,18,21,22,23,24]. The widely used “rule of six” describes the method of individualized doses based on patient weight, followed by dilution into a fix volume in a second syringe. This method was designed for quick dosing in emergency situations and before the availability of smart pumps. The “rule of six” is associated with high error rates, including tenfold dosing errors and inconsistent drug concentrations, which can potentially lead to complications such as hypoventilation or inadequate analgesia, through significant over- or under-dosing [22,23,24].

To decrease the ME rate, healthcare systems worldwide have begun to shift toward the use of standard concentrations (SCs) for continuous IV infusions. In 2004, the Joint Commission recommended the use of SCs for high-risk medications such as insulin and dopamine [25,26]. Various professional organizations, including ASHP [27,28], ISMP [25,29], RCPCH and NPPG [30], and initiatives like ePed in Sweden [31,32] have supported the development and implementation of SCs to reduce variability and increase safety.

Given the wide variability in weight and body composition among pediatric patients, it is challenging to balance precise dosing with appropriate fluid volumes using a limited number of SCs. Since this calculation often requires more steps than the calculation by the “rule of six”, many institutions are transitioning from outdated, less supportive systems to modern electronic prescription and administration tools that facilitate the use of SCs. Electronic prescribing and smart infusion pumps with drug libraries can support the selection of appropriate SC and calculating the corresponding volume and infusion rate [2]. Smart infusion pumps further enhance safety by displaying critical parameters such as dosage and rate, which enables verification and adjustment during the administration.

Several reviews have discussed various interventions in pediatric medication safety, but none have exclusively focused on the effect of SCs on IV infusion ME rates in experimental studies [9,33,34]. New experimental studies have evaluated the direct impact of implementing SCs on medication error rates. These are not captured in earlier reviews, which highlights the need for an up-to-date synthesis [2]. Given the global trend toward standardization and the ongoing debate about its effectiveness and feasibility, this review aims to systematically evaluate the impact of implementing SCs for IV infusions on ME rates in neonatal and pediatric settings.

## 2. Materials and Methods

### 2.1. Protocol and Registration

The guideline of Preferred Reporting Items for Systematic Reviews and Meta- Analyses (PRISMA 2020) was used to structure this systematic review [35,36]. This review was registered in PROSPERO (reg. no. CRD42024520237) and approved by the local ethics commission (EK 25-260).

### 2.2. Eligibility Criteria and Definitions

Studies that measured effects of implemented SCs for infusions on the ME rate in neonatal or pediatric healthcare institutions were included. The use of SCs was defined as the use of a few specified concentrations for each medication instead of the use of a variable dose in a defined volume.

In this systematic review, all studies in English language that evaluate the outcome of the implementation of SCs defined as MEs in neonatal and pediatric healthcare institutions were included. No study was excluded due to the simultaneous implementation of further interventions to reduce MEs. This review considered studies that evaluated interventions by comparing outcomes between an intervention group and a control group. Study designs were classified according to the framework of the Cochrane Effective Practice and Organisation of Care (EPOC) Review Group, which distinguishes randomized controlled trials (RCTs), controlled clinical trials (CCTs), controlled before-after studies (CBAs), and interrupted time series (ITS) [37]. Studies comparing outcomes between two patient groups with only one group exposed to the intervention and not meeting the classification of Cochrane EPOC Review Group were categorized as uncontrolled before-after studies (UBAs). The definition of MEs by Kaushal et al. as errors that occur during drug ordering, transcribing, dispensing, administering or monitoring is used in this systematic review [3]. The observed MEs were considered as outcome parameter and were not divided into further categories.

### 2.3. Information Source

The three databases MEDLINE, Embase and CINAHL were searched for studies describing the implementation of SC in pediatric healthcare settings. The references for all included articles were manually screened for additional studies.

### 2.4. Search Strategy

This systematic review analyses the time span from database inception up to 4th August of 2025. The search in the databases is based on a combination of five term groups: pediatric terms, standardization terms, concentration terms, infusion terms and error terms. The search strategy is adapted to each used database (Table S1). Backward and forward reference searching of all included articles completed the search [21,22,23,38,39].

### 2.5. Study Selection

Nine exclusion criteria were determined (Table S2). If no exclusion criterion applied to abstract and full text, the study was included in this systematic review. Two reviewers (L.W. and A.E.) screened all records for eligibility. The interrater agreement was calculated via Cohen κ [40].

### 2.6. Data Extraction and Calculation

For potentially eligible full texts, detailed data items were extracted. The data collection was examined independently by two reviewers (L.W., A.E.), and discrepancies were resolved by consensus. The collected data consisted of the publication details, study design, setting, methods, intervention, measured outcomes and results. Statistical tests were calculated using Microsoft Excel (Microsoft Corporation, Redmond, WA, USA).

### 2.7. Risk of Bias in Individual Studies

The risk of bias within the included studies was assessed using the ROBINS-I tool (Risk Of Bias In Non-randomized Studies of Interventions) [41]. This tool enables a systematic and standardized evaluation of potential bias in UBAs, evaluating the methodological quality of each study. Since only non-randomized studies were included in this review, the use of ROBINS-I tool was sufficient and no additional risk of bias assessment tools were required.

### 2.8. Summary Measures

Study outcomes were evaluated, with MEs reported in the UBAs used to calculate the relative risk reduction for individual or bundled interventions. If a study had insufficient data, the ratio of error before and after the implementation was calculated (Table 1). In the end, the results were summed up to estimate the effect of SCs on ME rates in neonatal and pediatric healthcare settings.

### 2.9. Synthesis of Results

Relative risk reductions were calculated by comparing pre- and post-intervention error rates, and statistical significance (*p* < 0.05) was assessed using Fisher’s exact test. Meta-Analyses are not eligible due to the heterogeneity of the included studies. The individual results, set in relation to their individual bias risk, were compared in a descriptive way.

### 2.10. Risk of Bias Across Studies

The risk of publication bias and time-lag bias of all included studies was estimated. Time-lag bias was assessed by calculating the time difference between data completion and electronic publication. The statistical significance of the time-lag bias was calculated by Mann–Whitney U-test (*p* < 0.05).

### 2.11. Additional Analyses

In addition to extracting data on the effect of SCs on ME rates, a qualitative analysis was conducted to identify other factors related to medication safety. Therefore, all included studies were reviewed in full text to extract information on implementation strategies and reported safety benefits beyond error reduction. Data extraction for the additional analyses focused on workflow, fluid volume management, staff-to-patient ratio, duration of medication administration processes, staff education as well as staff satisfaction and cost considerations of SCs in clinical practice.

**Table 1:** Included studies.

First Author (Country, Year)	Study Design, Study Center	Setting	Methods	Interventions	Measured Outcome	Results	Relative Risk Reduction
G. Larsen et al. (USA, 2005) [22]	UBA, single center	Pediatric hospital	Evaluation of reported medication errors in infusion therapies in 2002 (pre-implementation) and 2003 (post-implementation)	Standard concentrations for 32 medications smart syringe pump with modifiable drug library modified medication labels “train-the-trainer” strategy	Errors affecting standard concentrations and infusion pumps	The error rate was reduced from 3.1 to 0.8 per 1000 doses. (*p* < 0.001) Preparation errors in pharmacy were reduced from 0.66 to 0.16 per 1000 doses. 10-fold dosing errors were reduced from 0.41 to 0.08 per 1000 doses.	Overall: 74.2% (*p* < 0.001) Preparation: 75.8% 10-fold dosing: 80.5%
J. Bullock et al. (USA, 2006) [39]	UBA, single center	pICU	Analysis of medication error reports in a Medication Event Reporting System pre- and post-implementation	Standard concentrations for 27 intravenous medications Intensive education: One-on-one coaching, mentoring	Incorrect dosing and concentration	The error rate of incorrect dosage decreased from 26/50 incorrect orders to 7/28 incorrect orders. (*p* < 0.05) The error rate of incorrect concentration decreased from 6/26 orders with incorrect dosage to no incidents in 7 orders with incorrect dosage. (*p* < 0.05)	Dosing: 51.9% (*p* < 0.05) Concentration: 100% (*p* < 0.05)
S. Arenas-Lopez et al. (UK, 2017) [21]	UBA, single center	pICU	Analysis of morphine-related medication errors from the hospital incident reporting system over 8 years	Standard concentrations of morphine infusion for 4 weight bands (excluded nurse- and patient-controlled) Concentration individual storage and labeling Smart syringe pumps and electronic ward protocols	Morphine-related errors	126 errors related to morphine infusions were recorded. Drug errors related to morphine decreased from 45% to 2.2%. There were 24 prescription errors connected with standard concentrations in a total of 36 prescription errors. (22 of 24 did not result in patient harm.) Administration errors (e.g., programming errors or selecting the wrong syringe) occurred twice as frequently in standard concentrations (*n* = 46) compared to variable concentrations (*n* = 18, *p* = 0.025).	Morphine: 95.1% Administration RR: 2.0 → RRI 100% (*p* = 0.025)
A. Rashed et al. (UK, 2019) [23]	UBA, single center	Pediatric hospital	Analysis of morphine infusion (nurse- and patient-controlled analgesia) incident reports from the electronic reporting system (January 2013–December 2015)	Morphine standard concentrations for 3 weight bands (1 to 50 kg) The syringes were prepared by the pharmacy department. 3 months training, 8 months implementation	Risk of medication errors	54 failures occurred, 34 of them in the old system and 20 of them in the new system. Relative Risk Reduction is reported as 41.2% (*p* = 0.115). Details of error types are provided by the study.	Overall: 41.2% (*p* = 0.115)
M. Howlett et al. (Ireland, 2020) [38]	UBA, single center	pICU	Analysis of medication orders by a clinical pharmacist over 24 weeks across four time periods (Epochs 1–4)	Stepwise implementation: 1. Smart pumps with a drug library and standard concentrations for 4 weight bands 2. Electronic prescribing	Prescribing errors in medication orders Epoch 1: Before implementation Epoch 2: Directly after implementing SC and smart pumps Epoch 3: After implementing electronic prescribing Epoch 4: 1-year post-implementation.	3356 medication orders were reviewed by a pharmacist. 684 were infusion orders which caused 98 infusion-related prescribing errors: Epoch 1: 29.0% Epoch 2: 14.6% (*p* < 0.001) Epoch 3: 4.7% (*p* > 0.05) Epoch 4: 8.4% (*p* = 0.32) Paper generated error rate: Epoch 1: 78% Epoch 2: 97% Error rate in infusion: Epoch 1: 29% Epoch 4: 8.4%	Prescribing Epoch 1 → 2: 49.7% (*p* < 0.001) Epoch 1 → 4: 71.0% Paper-generated risk RR 1.24 → RRI 24.5%

UBA: uncontrolled before-after study, pICU: pediatric intensive care unit, RR: relative risk, RRI: relative risk increase.

## 3. Results

### 3.1. Study Selection

**Figure 1:**
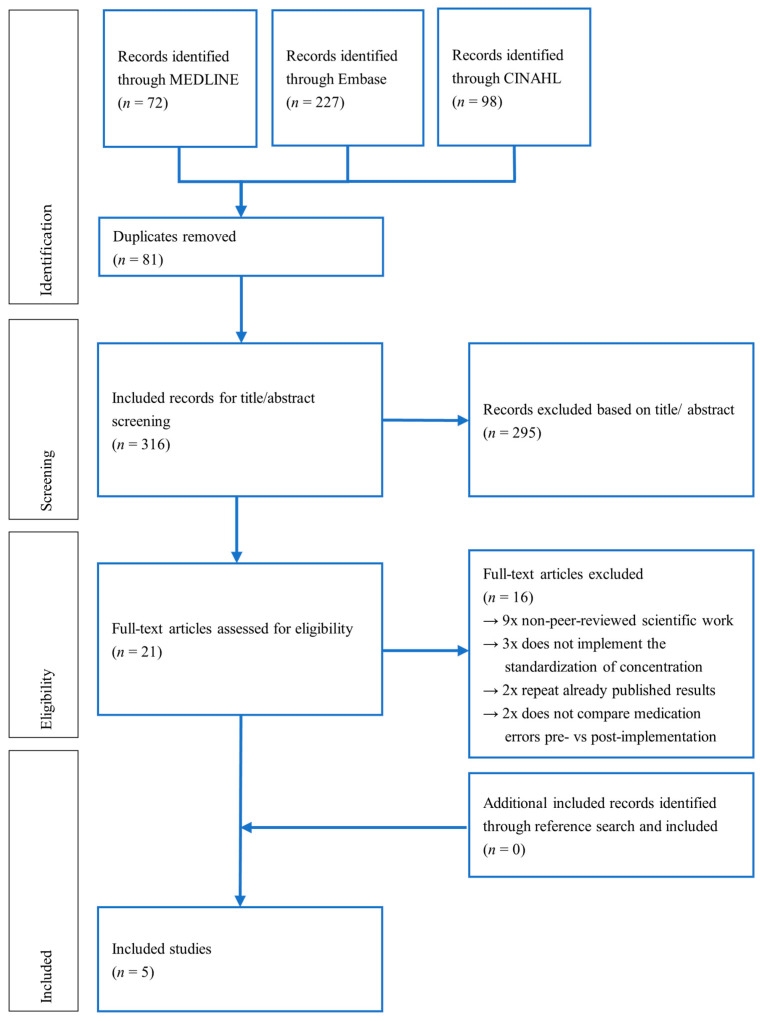
Study selection reported in PRISMA flowchart [36].

The search strategy was designed to analyze the effect of SCs on ME rates for intermittent and continuous infusions used in neonatal or pediatric settings. The results of the screening are shown in Figure 1. 72 results were found in MEDLINE, 227 in Embase and 98 in CINAHL. After removal of 81 duplicates 316 records were screened by title and abstract. 295 of the reviewed titles and abstracts applied to the predefined exclusion criteria, therefore 21 full-text articles remained. After the full-text screening, both reviewers agreed in the five included studies (Cohen κ = 1). Forward and backward searches of the included studies found 199 references, none of them were included as additional publications.

### 3.2. Study Characteristics

Five studies fulfilled all criteria [21,22,23,38,39]. All included studies were UBAs published between 2005 and 2020. The study characteristics are summarized in Table 2. Two studies were originated from the United States. Two studies were conducted out in the United Kingdom and one study in Ireland. Three studies were conducted in a pediatric intensive care unit (pICU) and two studies were carried out in pediatric hospitals. In three studies, the authors came from the same location, and in one study from two different cities within the same country. In one study, the authors were from two different countries. No RCTs, CCTs, CBAs and ITSs were included in this review. Three studies were UBAs that reported error rates as their primary outcome. Two studies were UBAs that compared the absolute number of MEs before and after implementation, without relating these numbers to the total number of medication orders. Therefore, a relative risk of medication errors cannot be calculated.

### 3.3. Risk of Bias Within Studies

Of the five included studies, four were assessed as having a critical risk of bias in at least one domain, resulting in an overall classification of high risk. One study was flagged for a serious risk in at least one domain. The main sources of bias arose from insufficient discussion of confounding factors and the simultaneous implementation of multiple interventions. The risk of bias estimated with ROBINS-I tool is shown in Table 3.

### 3.4. Synthesis of Results

**Figure 2:**
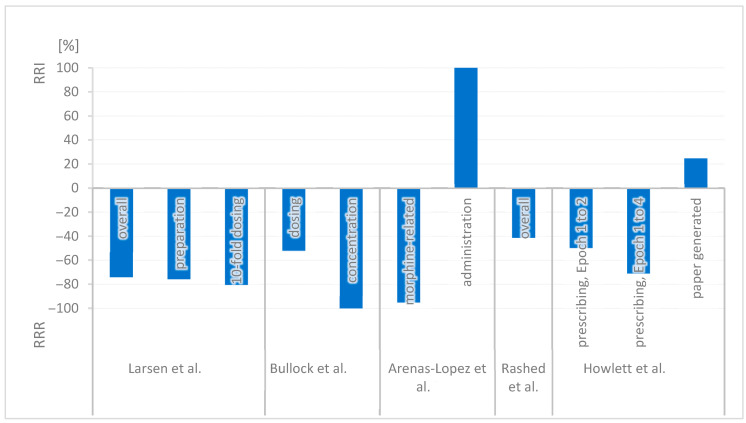
Relative Risk Reduction in Errors in Connection to SC (RRI: relative risk increase, RRR: relative risk reduction) [21,22,23,38,39].

A reduction in MEs was observed across the studies, often in combination with additional interventions such as electronic prescribing systems, smart pumps and drug libraries. The type of observed errors and the extent of error reduction varied depending on the clinical setting and the interventions performed. The results of the included studies are summarized in Table 1 and the relative risk reduction (RRR) and relative risk increase (RRI) are illustrated in Figure 2.

In 2005, Larsen et al. reported a decrease in overall ME rates after the introduction of SCs combined with smart pumps, a modifiable drug library, and improved labeling. The overall error rate dropped significantly from 3.1 to 0.8 per 1000 doses (RRR = 74.2%, *p* < 0.001), with a reduction in preparation errors (from 0.66 to 0.16 per 1000 doses, RRR = 75.8%) and ten-fold dosing errors (from 0.41 to 0.08 per 1000 doses, RRR = 80.5%) [22]. The *p*-value of the reduction in preparation errors and ten-fold dosing errors is not mentioned in the publication and cannot be calculated due to unknown absolute numbers.

In 2006, Bullock et al. observed a statistically significant reduction in dosing errors (from 52% to 25%, RRR = 51.9%, *p* < 0.05) and concentration errors (from 23% to 0%, RRR = 100.0%, *p* < 0.05). The implementation was accompanied by intensive education [39].

In 2017, Arenas-Lopez et al. found a relative reduction in morphine-related drug errors from 45% to 2.2% over 8 years (RRR = 95.1%). The *p*-value of the reduction is not mentioned in the publication and cannot be calculated due to unknown absolute numbers. Even if the overall error rate decreased, administration errors occurred twice as frequently with SCs (*n* = 46) than with variable ones (*n* = 18, RRI = 100.0%, *p* = 0.025), and 24 of 36 prescription errors of morphine infusions occurred were linked to the new SCs. The implementation of standard concentrations was combined with the implementation of smart syringe pumps and electronic ward protocols outlining the process from prescription to administration and specifying responsibilities. Individualized storage and labeling are intended to further enhance safety [21].

In 2019, Rashed et al. also reported a non-significant decrease in overall medication incidents of 41.2% (RRR = 41.2%, *p* = 0.115) after implementing SCs for morphine infusions prepared in the hospital pharmacy with intensive training. Certain error types, such as incorrect dose administration and wrong drug selection, were eliminated, while the rate of expiry date errors increased [23].

In 2020, Howlett et al. reported a significant error reduction after the implementation of SCs and smart pumps. Prescribing errors associated with infusions dropped from 29% to 14.6% (RRR = 49.7%, *p* < 0.001). Errors related to paper-based prescriptions increased (RRI = 24.5%). The *p*-value of the increase in prescription errors is not mentioned in the publication and cannot be calculated due to unknown absolute numbers. After the implementation of electronic prescription, the error rate dropped further to 8.4% one year post-implementation (RRR = 71.0%, *p* < 0.001) [38].

Despite differences in study design and implementation strategies, most studies reported significant reductions in ME after implementing SCs, particularly when combined with smart technologies and centralized preparation.

### 3.5. Risk of Bias Across Studies

Due to the high percentage of significantly positive results (60%), the possibility of publication bias cannot be excluded. In all studies, the time between end of data collection to publication could be calculated. Considering the significant studies, a median publication time of 24 months (interquartile range = 19–62) is determined. Considering only the studies with non-significant results, a median publication time of 27 months (interquartile range = 16–38) is determined. Using Mann–Whitney U-test a time lag bias cannot be confirmed (*p* > 0.05).

### 3.6. Additional Analyses

While the primary analysis of this review focused on the effect of SCs on ME rates, additional factors linked to medication safety were identified across the included studies. These additional factors are summarized in Table 4.

Two studies focused on the standardization of morphine infusions [21,23]. In one study, the SC was for morphine infusions used in nurse- and patient-controlled analgesia (N/PCA), while in the other N/PCA was excluded. The remaining studies implemented up to 32 different medications [22,38,39]. All studies mentioned the importance of multidisciplinary teams in the planning and implementation of SCs. The teams included physicians, pharmacists, nurses and informatics specialists. The involvement of all healthcare professionals in the planning of SCs was described as important for safe implementation [21]. Several studies highlighted how SCs simplify clinical workflows by reducing the need for bedside calculations. Arenas-Lopez et al. described a stepwise implementation of SCs, smart pump technology and electronic prescribing system, which eliminated the need for manual concentration, dilution and infusion rate calculations [21]. Larsen et al. noted that smart pump libraries could manage complex calculations, which reduces the cognitive burden on healthcare professionals and can potentially lower the risk of error [22]. Batch preparation of SC infusions was mentioned to reduce variability in drug concentrations and the risk of microbial contamination [21,23]. Rashed et al. measured a significant decrease in the overall medication administration time (11.9 ± 4.1 min to 3.7 ± 1.7 min). This gives healthcare professionals more time for patient care [23]. In pediatric and neonatal settings, fluid volume management is essential. Larsen et al. found that carefully selected SCs did not exceed acceptable fluid volumes for most patients [22]. This finding supports the feasibility of SCs even in vulnerable populations. Educational strategies were considered essential but varied across the studies. Arenas-Lopez et al. provided intensive support during the initial implementation period [21]. Bullock et al. offered various trainings, including staff meetings, lectures, electronic reminders and mentoring [39]. Larsen et al. employed a “train-the-trainer” approach to share knowledge effectively [22]. These training methods ensured the correct usage of SCs and contributed to long-term compliance. Howlett et al. stressed that even after the initial rollout, ongoing training is essential to maintain high compliance and safety standards [38]. In a survey conducted by Rashed et al., most healthcare professionals (95.2%) associated SCs with improved patient safety and 90.4% noted a reduction in drug delivery time [23]. Bullock et al. reported that their implementation was cost neutral [39]. Larsen et al. suggested that SCs could potentially reduce costs by reducing the number of preparations [22].

These additional findings highlight that various factors, such as interdisciplinary collaboration, workflow integration, ongoing education and technological infrastructure, will influence medication safety.

## 4. Discussion

### 4.1. Summary of Evidence

This systematic review aimed to evaluate the impact of SC infusions on ME rates in neonatal and pediatric settings. It complements previous reviews by providing an up-to-date synthesis of original studies with a clear focus on the effect of SCs on ME rates. Santesteban et al. as well as Nguyen et al. reviewed the types of MEs in neonatal care and highlighted preventive strategies, whereas the present review specifically focuses on the effect of SCs on ME rates across the entire neonatal and pediatric population in experimental studies [9,33]. While Santesteban et al. provided a comprehensive overview of ME types in NICUs, their review did not specifically isolate or quantify the impact of SCs on error rates [9]. The present review systematically synthesizes experimental evidence assessing the effect of SC implementation on ME rates in both neonatal and pediatric settings. Alharthi et al. examined the standardization of intravenous medicines and its implications for patient safety but did not evaluate the impact on MEs and was not limited to pediatric healthcare settings [34].

Several additional studies reported on the implementation of SCs but did not describe their impact on ME rates. They report that errors continued to occur after implementation, without collecting data in a controlled manner or providing baseline error rates before implementation [24,43,44,45,46]. Furthermore, some studies were excluded from this review as they addressed the implementation of SCs only from a theoretical perspective [47,48,49,50].

Five relevant studies were identified, all published between 2005 and 2020, which varied in design, setting, and implementation approaches. Across all included studies, a reduction and no increase in overall errors was reported, suggesting the potential of standardization as part of a broader medication safety strategy. As SC implementation was part of comprehensive intervention strategies, this error reduction was often measured in combination with other interventions such as smart pumps, electronic prescribing systems, or centralized preparation in the hospital pharmacy. Owing to heterogeneity in methodologies and definitions of MEs, the reported reductions in error rates attributed to SC implementation cannot be disentangled from concurrent interventions. Consequently, the error rates reported across the included studies are not reliably comparable [21,22,23,38,39].

Theoretical studies using risk analysis methods like Failure Mode and Effects Analysis (FMEA) and the National Patient Safety Agency (NPSA) risk score indicate potential safety advantages of SCs. Apkon et al. and Rashed et al. demonstrated a reduction in risk priority numbers across all steps of the medication process with SCs [23,47]. Perkins et al. and Barreto et al. similarly observed a decrease in risk scores when infusions were prepared in the hospital pharmacy rather than on the ward [49,50]. These theoretical studies are important to identify potential failure points and enhance the safety of the implementation process of SCs.

Training and education remain crucial for successful implementation of SCs. Implementing SCs can introduce new types of errors if staff are not adequately trained. Comprehensive education on prescription entry, infusion preparation, infusion rate calculation, smart-pump programming, and error recognition are essential [14,22]. Another potential error is the delayed detection of an occlusion in the infusion line, particularly when the infusion rate is low. Therefore, the alarm pressure threshold needs to be reviewed and the staff must be aware of this issue [51]. Introducing SCs can also lead to implementation challenges, including the selection of a limited number of concentrations that do not lead to fluid overload, especially in neonates. Studies indicate that, when carefully selected, SCs do not compromise fluid balance or safety, with only a few patients requiring other concentrations [44,45,50,52]. This contradicts the assumption that SCs are unsuitable for neonates and children.

Studies suggest that standardizing drug concentrations may contribute to the reduction in MEs, especially prescribing errors such as 10-fold dosing errors, and minimizes the need for bedside calculations, which are known to be error-prone [21,22,23,24]. Moreover, the risk of preparation errors decreases, especially when preparation takes place centrally in the hospital pharmacy rather than on the ward. It is well known that medication preparation on the ward is prone to errors due to various factors such as stress, interruptions and distractions [13,53]. Various studies stated that the centralization not only improves dosing accuracy but also reduces the risk of microbial contamination [21,23,46]. Similar findings were reported in multiple studies reviewed by Hedlund et al., although the generalizability of these results has been questioned due to the small sample sizes [53]. Nevertheless, it must be considered that the centralization needs an improved infrastructure in the hospital pharmacy, including additional staff and increased storage capacity [46]. Furthermore, pre-prepared syringes or commercially available ready-to-administer products can facilitate the workflow and increase the preparation safety even more [23].

Despite the largely positive outcomes, some studies report a paradoxical increase in MEs after implementing SCs [21,38,43]. Mitchell et al. considered the higher prescribing error rate observed after implementation to reflect improved detection (staff reports) rather than an actual increase in risk and the overall workflow was still regarded as more efficient, with enhanced staff confidence in dosing accuracy due to the elimination of complex calculations [43]. Arenas-Lopez et al. reported twice as many administration errors with SCs compared to weight-based concentrations, but these figures represent absolute counts without considering the total number of infusions or frequency of use. Therefore, a reported 100% increase cannot confirm a true rise in error rates [21]. Howlett et al. described an increase in paper-related errors without specifying the reasons. The proportion of these errors decreased after introducing electronic prescribing [38].

Therefore, the use of SCs with electronic prescribing has synergistic effects and enhances safety even more [38,44]. Electronically generated orders eliminate misinterpretations of handwriting and incomplete prescriptions. Together with smart pumps, SCs can be used easily. These smart pumps, when integrated with dose-checking drug libraries and barcode verification systems, create a closed-loop medication management system that significantly improves the plausibility and traceability of infusion administration [14,21,22,24]. In a closed-loop system user-friendly labeling with easily accessible infusion parameters is essential to avoid confusion during programming [21,22].

Using SCs, the medication process is improved by reducing the time required for medication prescription, preparation and administration [23,46,48,54]. Nurses and pharmacists benefit from simplified processes, which reduce cognitive load and lead to fewer interruptions [13]. Bullock et al. reported that the implementation was cost neutral [39]. Additionally, cost-effectiveness may be realized through reduced drug waste, extended stability, and fewer infusion preparation [22,47,55]. Nevertheless, the implementation may require investments, e.g., training, system updates and the purchase of technical equipment [56].

Finally, staff surveys further validate the advantages of SCs. Surveys consistently report higher satisfaction with the use of SCs, due to increased safety, faster drug delivery, and easier administration with smart pumps [23,38,47,54,57]. Nonetheless, the perception of benefit is closely tied to the users’ familiarity and knowledge with the new system, underlining the need for structured and ongoing training. The discussed results indicate potential benefits but also highlight context-dependent challenges of SCs. The advantages and challenges identified in this review are summarized in Table 5.

### 4.2. Strength and Limitations

This review provides a focused synthesis of studies considering the impact on the effect of SC infusions on reported MEs in pediatric and neonatal settings. The inclusion of studies from multiple countries increases the generalizability of the findings. However, the strength of the evidence is limited by heterogeneity in study design, clinical settings, and the bundle of interventions. Moreover, only a very limited number of studies is available, which further weakens the robustness of the evidence base. All studies were uncontrolled before-after-designs with small sample sizes and a high risk of bias. ME data were often based on incident reporting, which is known to be underreported. Differences in reporting culture and detection systems between institutions may contribute to the variability of reported ME rates across studies [58]. In some cases, small post-implementation groups further limited statistical validity.

In addition, SCs were rarely implemented in isolation. Other interventions such as smart pumps, electronic prescribing, and staff training were introduced at the same time, which makes it difficult to relate outcomes only to SCs. Increased awareness of MEs after implementation may also have influenced error detection and reporting. While publication bias could not be statistically confirmed, the high number of positive findings suggests that it cannot be fully excluded.

Larger, multicenter experimental studies are needed to provide more robust data to clarify the true impact of SC infusions on medication safety. Studies should be conducted in diverse clinical settings with standardized outcome reporting. In addition to error rates, future studies should examine long-term effects, including the benefits for clinical workflow and patients. Finally, national or international harmonization of SCs should be explored. This could enhance patient safety, reduce variability, and generate cost savings even more.

## 5. Conclusions

This systematic review suggests that the implementation of SC infusions may help to reduce MEs in pediatric and neonatal settings. They enable the use of electronic prescribing systems, facilitate hospital pharmacy-centered preparation with higher accuracy and hygiene standards, and can be prepared in advance since preparation is no longer required on a patient-specific basis, which can improve workflow efficiency. SCs can be administered safely with smart pumps. Smart pump technologies can automatically calculate infusion rates, apply soft and hard dose limits, and verify medication parameters using barcode scanning. However, the available evidence is not only limited by small sample sizes, high risk of bias, and heterogeneity in study designs, but also by the fact that only a very small number of studies exists. This considerably limits the reliability of the conclusions. SC alone may not eliminate all errors and may introduce new risks, especially during implementation. Therefore, healthcare institutions should implement SCs carefully and in combination with supportive technologies, staff training, system redesign, and continuous monitoring to maximize their safety benefits. Further high-quality studies such as RCT and long-term studies are needed to confirm or refute the positive effect of SCs on the reduction in MEs in pediatric and neonatal settings.

## Figures and Tables

**Table 2:** Study Characteristics.

Study Characteristic	Number of Included Studies
**Study design**	
RCT, CCT, CBA, ITS	0
UBA	5
**Authorship**	
International	1
Same country	1
Same city	3
**Setting**	
Pediatric hospital	2
pICU	3
**Country**	
Ireland	1
UK	2
USA	2

RCT: randomized controlled trial, CCT: controlled clinical trial, CBA: controlled before-after study, ITS: interrupted time series, UBA: uncontrolled before-after study, pICU: pediatric intensive care unit.

**Table 3:** Risk of Bias Within Studies estimated with ROBINS-I tool.

	Bullock et al. [39]	Rashed et al. [23]	Larsen et al. [42]	Howlett et al. [38]	Arenas-Lopez et al. [21]
Bias due to confounding	Serious risk	Critical risk	Critical risk	Critical risk	Critical risk
Bias in selection of participants	Low risk	Low risk	Low risk	Low risk	Low risk
Bias in classification of interventions	Low risk	Low risk	Low risk	Low risk	Low risk
Bias due to deviations from intended interventions	Low risk	Low risk	Low risk	Low risk	Low risk
Bias due to missing data	Moderate risk	Low risk	Moderate risk	Low risk	Low risk
Bias in measurement of outcomes	Low risk	Moderate risk	Moderate risk	Low risk	Low risk
Bias in selection of reported results	Moderate risk	Moderate risk	Moderate risk	Moderate risk	Moderate risk
Overall bias	Serious risk	Critical risk	Critical risk	Critical risk	Critical risk

**Table 4:** Additional Factors Influencing Medication Safety in Connection to Standard Concentration (SC).

Additional Factors	Description and Effect	Mentioned in
**Implementation Team**	A multidisciplinary team, including physicians, nurses, pharmacists and medical informaticians in planning and implementation	Arenas-Lopez et al. [21]
**Workflow**	Reduction in bedside calculations, when combining with smart pumps and electronic prescribing systems	Arenas-Lopez et al. [21], Larsen et al. [22]
	Central/batch preparation to reduce deviations in concentrations and microbial contamination risk	Arenas-Lopez et al. [21], Rashed et al. [23]
	Measured reduction in medication process time	Rashed et al. [23]
**Fluid Volume Management**	With well-chosen SC, in most patients, fluid overload must not be expected	Larsen et al. [22]
**Staff Education Methods**	Initial and ongoing to guarantee a safe implementation process. (e.g., lectures, mentoring, train-the-trainer, electronic reminders)	Arenas-Lopez et al. [21], Bullock et al. [39], Larsen et al. [22], Howlett et al. [38]
**Staff Satisfaction**	Survey resulted in a perception of improved safety and time savings	Rashed et al. [23]
**Cost Considerations**	Cost-neutral implementation costs to a potential reduction in costs	Bullock et al. [39], Larsen et al. [22]

**Table 5:** Advantages and Challenges of Standard Concentrations.

	Advantages	Challenges
**Medication Safety**	Reduces prescribing errors (e.g., 10-fold dosing errors) [21,22,23] Minimizes bedside calculations [21,22,23,24,44]	New types of errors may occur if staff are not trained properly [22] In low infusion rates an occlusion of the infusion line is noticed delayed [51]
**Preparation Accuracy**	Reduces preparation errors, especially with centralized preparation [21,23,45,53] Less risk of microbial contamination when prepared centralized [21,23,46]	Requires infrastructure for central preparation [46] or commercially available products
**Workflow Efficiency**	Faster prescribing, preparation, and administration [23,46,48,54] Reduced cognitive load and interruptions for healthcare professionals [13,23,54]	Selection of concentrations must avoid risks like fluid overload (esp. in neonates) [44,45,50,52] If a patient needs an individualized concentration, the medication process is no longer a routine process which makes it more error prone [54] Risk of selection errors increases with number of different concentrations available in drug library [22,23]
**Technology Integration**	Synergistic with electronic prescribing, smart pumps, barcode systems [22,24,38,46]	Technical and organizational integration can be complex [21,44,51]
**Risk Management**	Decreases risk priority numbers across medication process in risk analysis methods (FMEA, NPSA score) [23,47,49,50]	Requires careful implementation planning to identify potential failure points [14,22,23]
**Staff Perspective**	Increases confidence and satisfaction [23,38,47,54,57] Perceived safety, faster drug delivery [23,38,47,54,57]	User acceptance depends on familiarity; therefore, training is crucial [22,38]
**Cost and Resources**	Potential cost-effectiveness (less waste, less preparations, extended drug stability) [22,39,47,55] Implementation was cost-neutral in some settings [39]	Initial training and system updates may require investment [56]

FMEA: Failure Mode and Effects Analysis, NPSA: National Patient Safety Agency.

## Data Availability

All data is included in this systematic review.

## Supplementary Materials

The following supporting information can be downloaded at: https://www.mdpi.com/article/10.3390/jcm14227965/s1, Table S1. Search terms used for each database, Table S2. Exclusion criteria for abstracts and full texts.

## Abbreviations

The following abbreviations are used in this manuscript:

ADE	adverse drug event
CBA	controlled before-after study
CCT	controlled clinical trial
EPOC	Cochrane Effective Practice and Organisation of Care
FMEA	Failure Mode and Effects Analysis
ITS	interrupted time series
IV	intravenous
ME	medication error
N/PCA	nurse- and patient-controlled analgesia
NICU	neonatal intensive care unit
NPSA	National Patient Safety Agency
pICU	pediatric intensive care unit
PRISMA	Preferred Reporting Items for Systematic Reviews and Meta-Analyses
RCT	randomized controlled trial
ROBINS-I tool	Risk Of Bias In Non-randomized Studies of Interventions
RRI	relative risk increase
RRR	relative risk reduction
SC	standard concentration
