# Supplementary material for: The Effect of Standard Concentration Infusions on Medication Errors in Neonatal and Pediatric Healthcare Settings: A Systematic Review
Source: J Clin Med. 2025 Nov 10;14(22):7965. doi: 10.3390/jcm14227965 (PMC12653657; doi:10.3390/jcm14227965)
Supplement: Supplementary file 1 [file jcm-14-07965-s001.zip › jcm-3931166-supplementary.pdf]

Table S1. Search terms used for each database.

Database	Search strategy
**PubMed/ MEDLINE**	("pediatr*"[Title/Abstract] OR "paediatr*"[Title/Abstract] OR "infant*"[Title/Abstract] OR "neonat*"[Title/Abstract] OR "newborn*"[Title/Abstract] OR "toddler*"[Title/Abstract] OR "child*"[Title/Abstract] OR "adolescen*"[Title/Abstract] OR "teen*"[Title/Abstract] OR "youth*"[Title/Abstract]) AND ("standard*"[Title/Abstract]) AND ("concentration*"[Title/Abstract]) AND ("infusion*"[Title/Abstract] OR "iv"[Title/Abstract] OR "i.v."[Title/Abstract] OR "intraven*"[Title/Abstract]) AND ("error*"[Title/Abstract])
**Embase**	('pediatr*':ti,ab OR 'paediatr*':ti,ab OR 'infant*':ti,ab OR 'neonat*':ti,ab OR 'newborn':ti,ab OR 'toddler*':ti,ab OR 'child*':ti,ab OR 'adolescen*':ti,ab OR 'teen*':ti,ab OR 'youth*':ti,ab) AND 'standard*':ti,ab AND 'concentration*':ti,ab AND ('infusion*':ti,ab OR 'iv':ti,ab OR 'i.v.':ti,ab OR 'intraven*':ti,ab) AND 'error*':ti,ab
**CINAHL**	(pediatr* OR paediatr* OR infant* OR neonat* OR newborn* OR toddler* child* OR adolescen* OR teen* OR youth*) AND standard* AND (concentration* OR dose* OR dosing* OR dosa*) AND (infusion* OR iv OR i.v. OR intraven*) AND error*

Table S2. Exclusion criteria for abstracts and full texts.

Exclusion Criteria for abstracts and full texts
No author and/or no abstract available
Non-peer-reviewed scientific work, e.g. conference abstract, poster presentation, doctoral thesis
Does not implement the standardization of concentration
Does not deal with intermittent or continuous infusion
Does not compare medication errors pre- vs. post-implementation
Preparation is a mixture of more than one active component in a diluent
Full text is not in English language
Repeats already published results
Does not take place in a health care system
